# Improving Perovskite/CIGS Tandem Solar Cells for Higher Power Conversion Efficiency through Light Management and Bandgap Engineering

**DOI:** 10.1021/acsami.5c15458

**Published:** 2025-09-25

**Authors:** Guillermo Farias-Basulto, Thede Mehlhop, Nicolas J. Otto, Tobias Bertram, Klaus Jäger, Stefan Gall, Nikolaus Weinberger, Rutger Schlatmann, Iver Lauermann, Reiner Klenk, Emil List-Kratochvil, Christian A. Kaufmann

**Affiliations:** † 28340 Helmholtz-Zentrum Berlin für Materialien und Energie GmbH, Hahn-Meitner-Platz 1, Berlin 14109, Germany; ‡ HTW Berlin - University of Applied Sciences, Wilhelminenhofstr. 75a, Berlin D-12459, Germany; § Zuse Institute Berlin, Takustraße 7, Berlin 14195, Germany; ∥ Universität Innsbruck, 27255 Institut für Konstruktion und Materialwissenschaften, Technikerstraße 13, Innsbruck 6020, Austria; ⊥ Humboldt-Universität zu Berlin, Institut für Physik, Institut für Chemie, Zum Großen Windkanal 2, 12489 Berlin, Germany; # Center for the Science of Materials Berlin, Zum Großen Windkanal 2, Berlin 12489, Germany

**Keywords:** photovoltaic, thin-film, solar Cell, CIGS, perovskite, tandem, record

## Abstract

Perovskite and chalcopyrite materials are excellent absorbers for highly efficient, all-thin-film tandem solar cells. This work presents a certified world record for such a device, achieving a power conversion efficiency of 24.6 ± 1.1% under steady-state conditions. The best *IV* parameters extracted from certified current–voltage measurements presented a short-circuit current density of around 19.3 mA/cm^2^, an open-circuit voltage of 1.765 V, and a fill factor of 71.8%. In comparison to our previous record, the current density improved considerably, mainly due to the lowering of the bandgap of the bottom subcell and the improved optics of the top perovskite cell.

## Graphical abstract

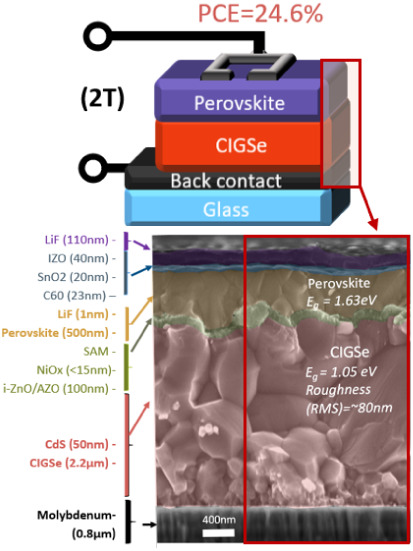

## 1 Introduction

To this day, hybrid metal halide perovskites are among the most investigated material classes in solar energy research due to their cost-effective processing and the high efficiencies achieved in an outstandingly short period of time.^1^ Perovskites can be deposited at low temperatures and can therefore be placed onto narrow-bandgap bottom cells to fabricate photovoltaic tandem devices. Perovskites offer a tunable wide bandgap near the optimal range for the top cell of perovskite-based tandem solar cells, which have achieved (in the case of perovskite/Si tandems) power conversion efficiencies above 30% (up to 34.6%),^2^ exceeding the Shockley–Queisser limit for single-junction devices.^3,4^

Chalcopyrite materials, such as CIGS, namely Cu­(In,Ga)­Se_2_, are tunable for narrow bandgaps (i.e., 1.0 – 1.3 eV^5,6^ and have proven highly efficient as single-junction solar cells (e.g., a record of 23.6%),^7^ which makes them ideal partners for perovskites to achieve highly efficient all-thin-film tandem devices. As CIGS can be grown through coevaporation processes,^8,9^ it becomes possible to continuously control the deposition rate of the various elements, which enables narrow effective bandgaps while maintaining a bandgap grading to minimize interface and back-contact recombination.^10^ This is a competitive advantage for double- and triple-junction devices, as the narrow bandgap becomes crucial for higher efficiencies.^6,11–13^ In addition, the importance of optical improvements to reach high efficiencies (>30%) was emphasized in the work of our previous perovskite/CIGS tandem record by Marko Jost et al.^14^ Therefore, bandgap engineering of CIGS^11^ and optical improvement^14^ are extremely important for highly efficient perovskite/CIGS solar cells.

In this work, we present a world record for a monolithically grown two-terminal (2T) perovskite/CIGS tandem solar cell, which achieved a certified steady-state performance of 24.6% ± 1.1% with an active area of 1.105 ± 0.067 cm^2^. This was primarily achieved by lowering the bandgap of the bottom cell and through optical improvements. It is worth mentioning that this device achieved a noncertified power conversion efficiency (PCE) of 27%. Figure 1 presents an optical image of the device.

**Figure 1:**
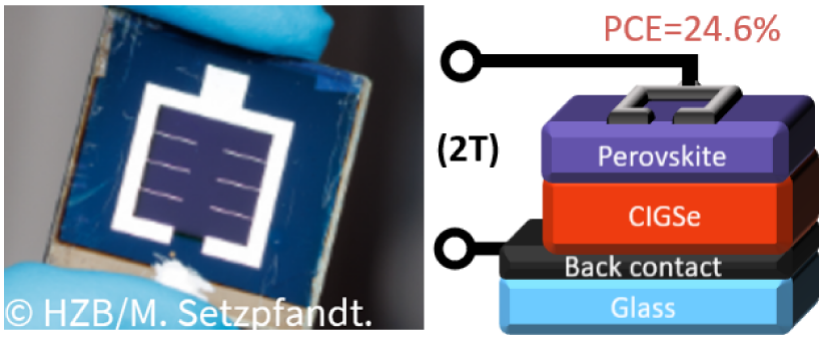
Optical image of the new record cell with a simplified representation of the two-terminal tandem.

## 2 Methods and Materials

The bottom CIGS cell was fabricated, as previously described, using a three-stage coevaporation process,^14,15^ where a CIGS absorber of about 2.2–2.4 μm thickness was grown on soda-lime glass coated with molybdenum. Rubidium fluoride was used as a postdeposition treatment.^15,16^ After the absorber layer, the buffer layer was deposited using a chemical bath deposition of cadmium sulfide (CdS) with a thickness of 50 nm, followed by two sputtered layers of intrinsic and aluminum-doped zinc oxide (i-ZnO/Al:ZnO) with a total thickness of ∼100 nm, serving as the electron transport layer (ETL) for the bottom cell. The total root-mean-square roughness of the bottom cell, measured through confocal microscopy, was ∼80 nm.

To decrease the bandgap of the bottom cell, the Ga in-depth profile, characteristically observed in CIGS devices, was modified simply by lowering the temperature of the substrate during the second and third stages of the 3-stage deposition process from nominally 530 °C down to 490 °C. By means of glow discharge optical emission spectroscopy (GDOES), the depth-dependent elemental composition of the differently grown CIGS absorbers was measured.^17,18^ From these GDOES measurements, the Ga/(In + Ga)-ratio (GGI) profiles along both CIGS film’s depths were calculated.

Figure 2 shows bandgaps as a function of depth, calculated from the GGI profiles of representative bottom cells fabricated at the two previously mentioned process temperatures: our process nominally at 530 °C in black (as in our previous record from 2020) and 490 °C in red (for the record cell in 2024). The fluctuation in the reproducibility of the in-depth composition is attributed to slight variations in the positioning of the thermocouple used for substrate temperature measurement. The bandgap grading of our current record material from the same process batch is highlighted in green. The minimum bandgap obtained from these curves is shown to facilitate the visualization of the bandgap decrease from about 1.08–1.1 eV to 1.05–1.06 eV, respectively.

**Figure 2:**
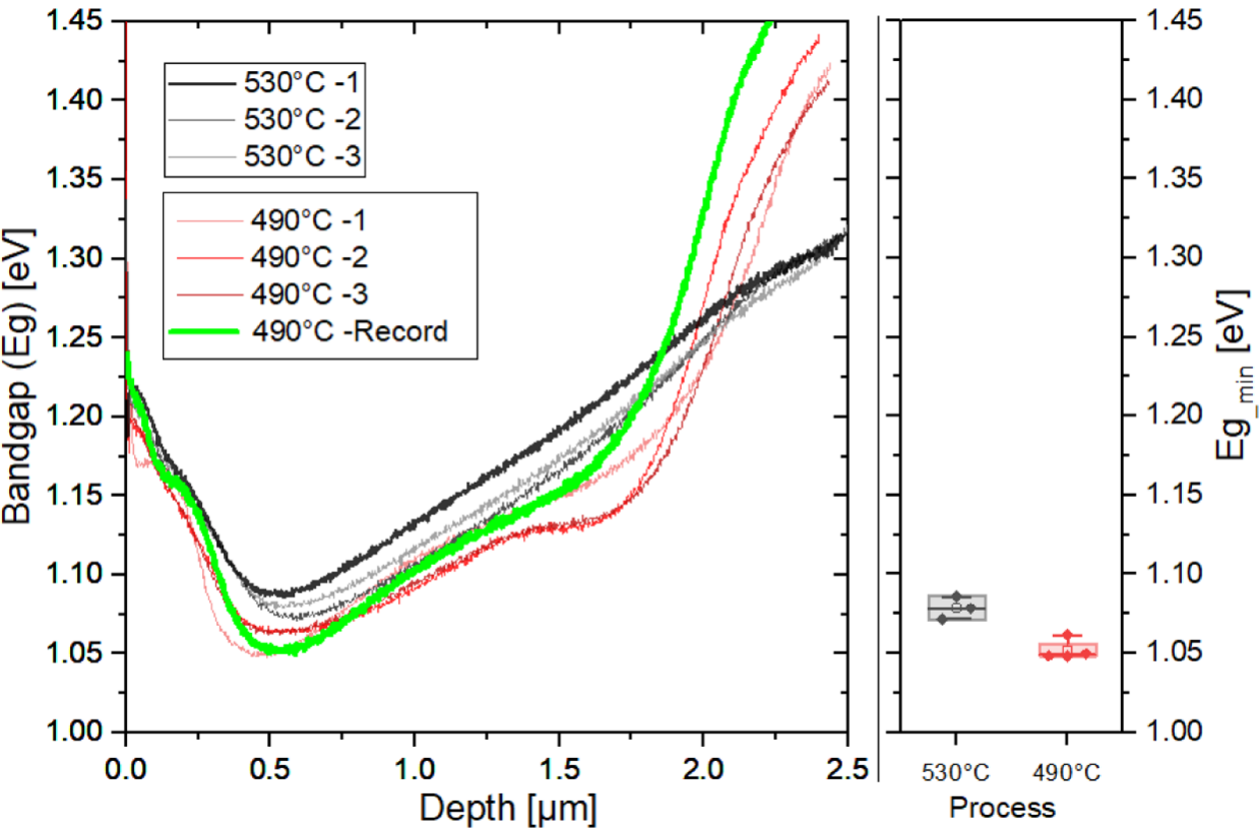
Bandgap profile of reference bottom cells from exemplary baseline processes for higher and lower bandgaps. The reference process from our record material is highlighted in green.

A layer of nickel oxide (NiO_*x*_) was included at the interface of the perovskite top subcell and the bottom CIGS subcell. A solution containing NiO_*x*_ nanoparticles (5 mg/mL) was spin-coated on the bottom cell as part of the hole transport layer (HTL). To prepare the bottom cells for HTL deposition, the bottom cell was cleaned with nitrogen to remove dust particles and placed in a UV-ozone treatment for 15 min. Prior to deposition, the NiO_*x*_ solution was conditioned using a sequence of 15 min in an ultrasonic bath, solution filtering (0.2 μm PTFE), and further ultrasonic bath treatment for another 5 min. After the bottom cell surface cleaning and activation by the ozone treatment, the NiO_*x*_ nanoparticle solution was spun at 2000 rpm for 30 s and annealed at 150 °C on a hot plate for 15 min. This resulted in a NiO_*x*_ layer thickness of only 2.8 ± 0.9 nm, as measured by wavelength dispersive X-ray fluorescence (WDXRF). Hence, assuming the size of the nanoparticles is larger than 2 nm, it is likely that a nonuniform layer was deposited. Further details on the precision of the WDXRF measurements are described in Supporting Information. After the deposition of NiO_*x*_, 2PACz, as a self-assembled monolayer (SAM), was spin-coated onto the sample. The 2PACz precursor was dissolved in ethanol (0.0033 mol/L) and placed in an ultrasonic bath for 10 min at room temperature. The substrate was blown with nitrogen to remove dust particles, followed by the deposition of 100 μL of the SAM solution onto the substrate. The solution was first spin-coated at 1000 rpm for 10 s, followed by 30 s at 3000 rpm. After spin-coating, the substrates were placed on a hot plate at 100 °C for 10 min in a nitrogen atmosphere.

Similarly to our previous perovskite/CIGS tandem record, a triple-cation perovskite was used. However, its composition was altered slightly to produce a narrower bandgap perovskite of 1.63 eV instead of 1.68 eV (i.e., Cs_0.05_(FA_0.83_MA_0.17_)_0.95_Pb­(I_0.83_Br_0.17_)_3_). The perovskite solution was spun for 5 s at 3000 rpm and then for 35 s at 3500 rpm. After the spinning started, antisolvent was also deposited onto the substrate and was later annealed on a hot plate for 30 min at 100 °C. A more detailed description of the perovskite preparation is provided in Supporting Information. A passivation layer of lithium fluoride (1 nm LiF) and an ETL of fullerene C_60_ (23 nm) were evaporated onto the perovskite. As the window layer, a combination of SnO_*x*_, indium-doped ZnO (IZO), and LiF was used. The SnO_*x*_ layer was deposited using an atomic layer deposition (ALD) process (20 nm), followed by a ∼40 nm layer of sputtered IZO and 500 nm of silver, which was evaporated as the top contact through a mask, defining an active area of 1.105 cm^2^. Finally, a ∼110 nm thick LiF layer was evaporated as an antireflection coating. The complete structure of the tandem cell is visualized in Figure 3.

**Figure 3:**
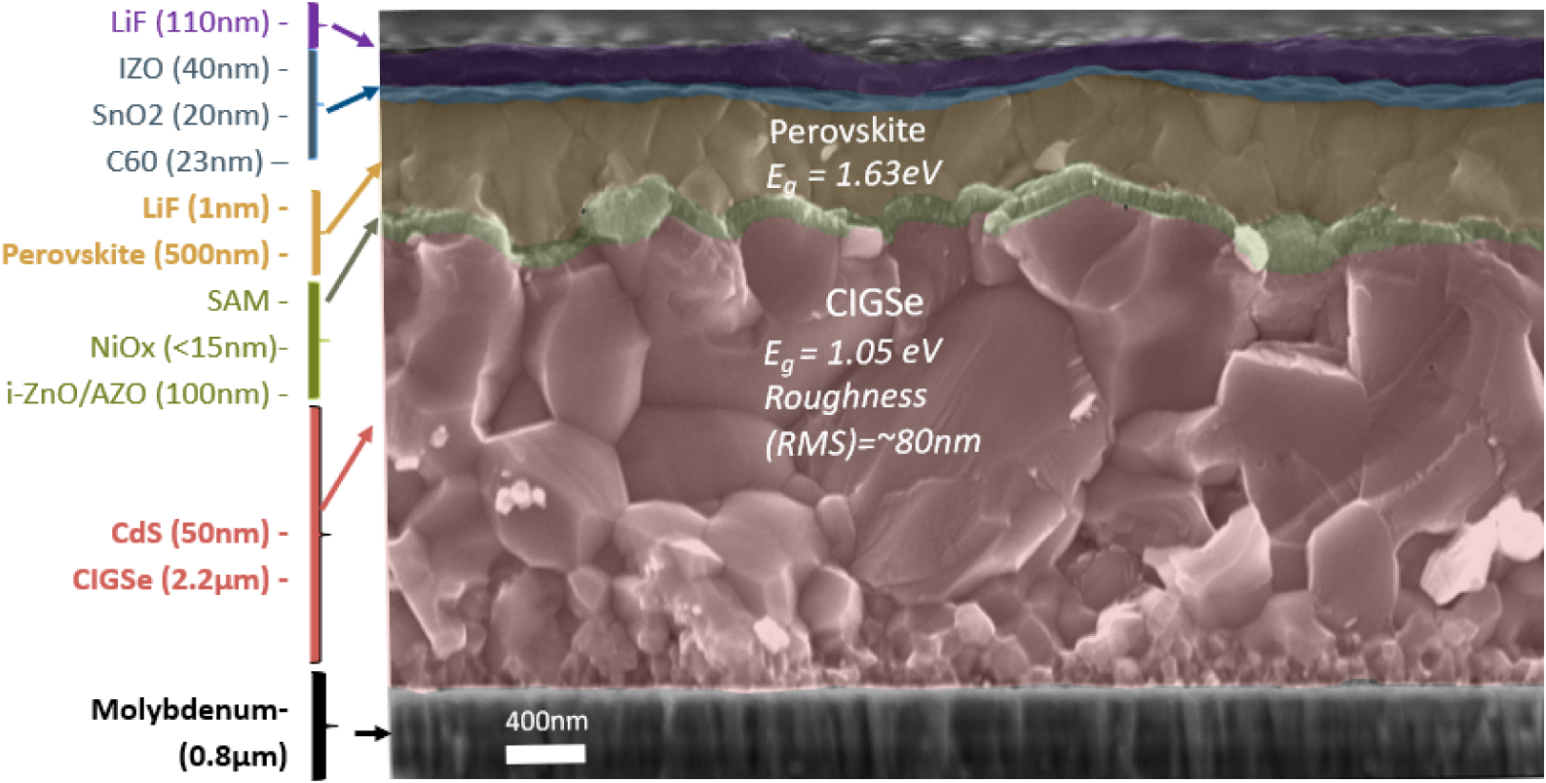
Scanning electron microscopy (SEM) image of the cross-section of a perovskite/CIGS tandem solar cell before encapsulation.

## 3 Results

As previously stated, optical performance is one of the limitations to achieving 30% efficiency in perovskite/CIGS tandem solar cells. Figure 4 shows the results of optical simulations that we performed to study how reducing the nominal IZO thickness from 100 to 40 nm affects the optical performance of the solar cell. We performed this simulations with GenPro4, which is based on the net radiation method.^19^ The simulations show that reducing the IZO thickness increases the current densities in perovskite and CIGS each separately by 0.6 mA/cm^2^. The total parasitic absorption in IZO is reduced by 0.74 mA/cm^2^. Further, reducing the IZO thickness also reduces reflective losses by 0.6 mA/cm^2^. Details about the simulations are given in Supporting Information Section S1.

**Figure 4:**
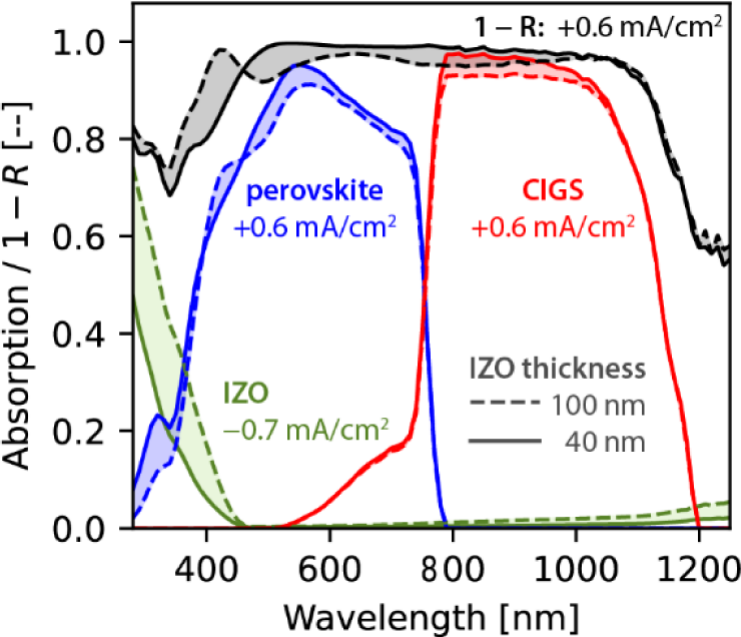
Optical simulations of perovskite/CIGS solar cells. The sign of the current densities shown indicates the change when the IZO thickness is reduced from 100 to 40 nm. Details on the simulations are given in Supporting Information Section S1.

The effects of bandgap grading and optical improvements are visible in Figure 5, which presents both the top (blue) and bottom cell (red) external quantum efficiency (EQE) curves, from which the bandgaps and the total available current were extracted. Using the first derivative of the individual curves, an approximation of the bandgap was made, obtaining 1.632 and 1.042 eV for the top and bottom cells, respectively. These values accurately match the values expected from the chemical composition in the perovskite, as well as from GDOES measurements of the reference bottom cells (maximum deviation of 8 meV). The available current is extracted using the spectral response (SR) together with the reference spectrum AM1.5G.^20,21^ Both the EQE and SR measurements were certified and provided by CalLab Fraunhofer ISE.^7,21–25^ Note that for the comparison in Figure 5, we have employed the certified EQE values from the previous record, which are different from the in-house measured values presented by Marko Jost et al.^14^

**Figure 5:**
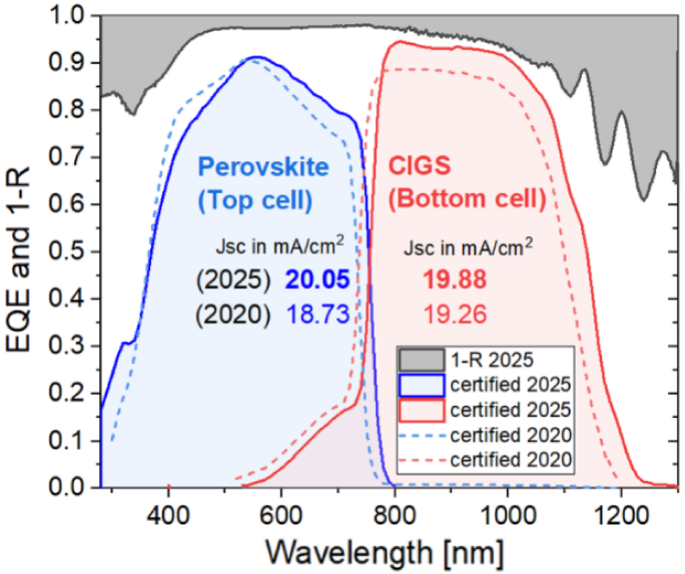
External quantum efficiency curves of the perovskite/CIGS tandem from certified measurements.

As the current from the bottom cell is lower, the tandem device is now bottom-limited. It is worth mentioning that the roughness of the CIGS might have contributed even further to the harvesting of photons within the device. Moreover, in addition to the photogenerated current gain, the top cell also achieved a larger current density, possibly due to the lowering of the bandgap together with a better cell design using a “finger-grid” described by Mariotti et al.^26^

According to our calculations from certified SR measurements (both previous and our current record), the current density of both bottom and top subcells increased from 19.26 to 19.88 mA/cm^2^ and from 18.73 to 20.05 mA/cm^2^, respectively. Therefore, not only was the overall photogenerated current increased, but also the current mismatch at AM1.5G decreased (i.e., from 0.53 to 0.17 mA/cm^2^). For visualization, Figure 6 presents the number of converted photons from available photons in the standard AM1.5G solar spectrum at each individual subcell of the 24.6% efficient tandem device.

**Figure 6:**
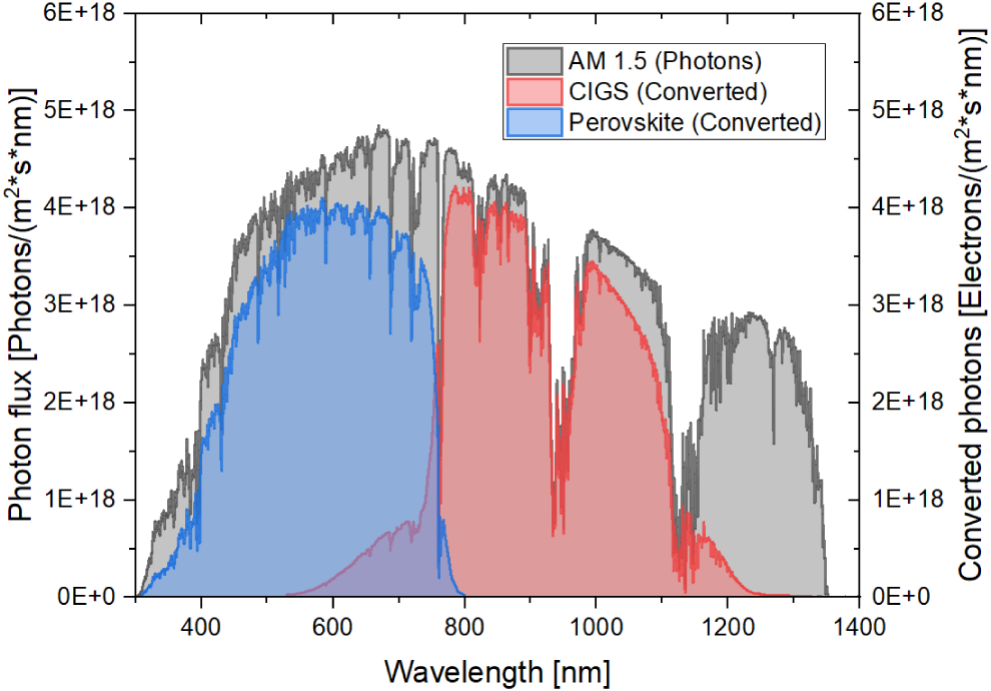
Number of photons converted from available photons in the standard solar spectrum AM1.5G in each subcell of the new record according to the certified spectral response measurement.

The current–voltage (*IV*) certification measurement of the perovskite/CIGS tandem was performed at CalLab Fraunhofer ISE.^7,21–25^ The certificate is presented in Figure 7A, showing the *IV* measurements (i.e., *V*_oc_ to *I*_sc_ and *I*_*sc*_ to *V*_oc_) performed after a 40 min light-soak treatment. The maximum *IV* parameters obtained from the certified measurements were a PCE of 24.5%, an MPP of 27.0 mW, a *J*_sc_ of 19.29 mA/cm^2^, a *V*_oc_ of 1.765 V, and an FF of 71.8%. As shown in Figure 7B, the certified record was obtained during a steady-state measurement lasting over 500 s, where a PCE of 24.6 ± 1.1%, visible also in the NREL chart for world record efficiencies,^1^ was achieved. The *IV* parameters, as well as the record steady-state measurement, are embedded as a table in Figure 7A.

**Figure 7:**
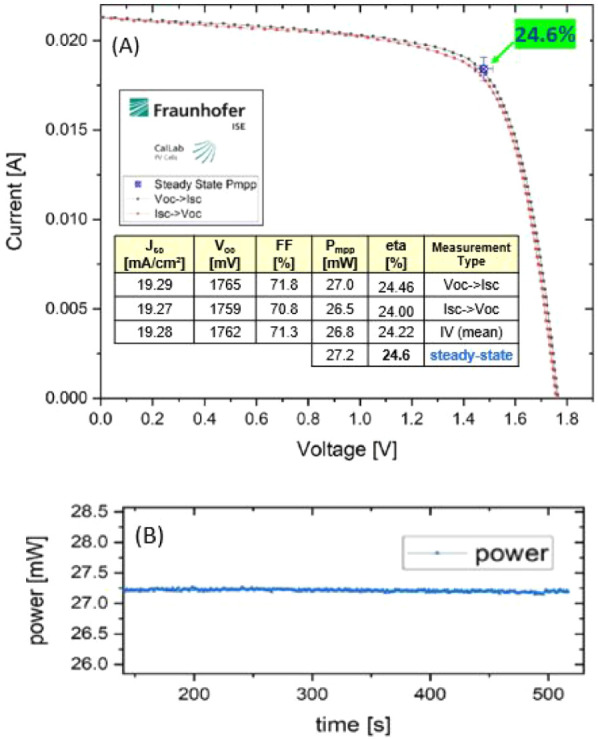
Certified measurements of the perovskite/CIGS tandem performed by CalLab Fraunhofer ISE with an active area of 1.105 cm^2^. (A) Current–voltage measurements from *V*_oc_ to *I*_sc_ and vice versa. (B) Steady-state MPP-tracked measurements.

## 4 Discussion

It is clear that we successfully eliminated the top cell limitation present in the previous record cell. Comparing the EQE, its current has increased by ≈1.3 mA/cm^2^. The optical model suggests a gain of 0.6 mA/cm^2^ from the reduced IZO thickness. It nominally follows that the reduced top cell bandgap contributes about 0.7 mA/cm^2^ to the current gain. The reduced top cell bandgap by itself would have resulted in a loss of bottom cell current; however, the reduced IZO thickness and reduced bottom cell bandgap counteract this loss, leading to an overall improvement in the bottom cell current as well. The roughness of the CIGS improves its current, whereas its influence on top cell current is negligible (Figure SI.2).

While the above indicates the importance of reducing the IZO thickness, there might have been a reduction in fill factor due to the increased sheet resistance of the thinner IZO (120.2 vs 48.7 Ω/□ as measured for reference films on glass). However, the new cell design includes a modified grid with fingers, which makes the cell less susceptible to that TCO resistance.^26^ Indeed, the fill factor of the new cell is even slightly higher.

The increase in the limiting current from 18.73 (old, top) to 19.88 (new, bottom) is not fully reflected in the jV data of the old and new devices, respectively. One problem in comparing the various numbers is the (meta)­stability of the device. Our initial noncertified measurement of 23.37% PCE with a *J*_sc_ 19.84 mA/cm^2^ improved after a series of light-soaking preconditioning up to about 27.01% PCE with a *J*_sc_ of 20.08 mA/cm^2^, which are closer to the certified-SR value. However, upon receipt of the sample back from certification, the sample was notably degraded. Therefore, the discrepancy found in *J*_sc_ is likely a result of the metastability and possible degradation of the device due to preconditioning. However, both certified-SR and certified IV-measurement *J*_sc_ values fall within our various in-house measurements, which can be found in Supporting Information Section S.3

Another difficulty in comparing jV and EQE current densities lies in the 2-terminal configuration, which does not allow direct electrical access to the individual cells. In the presence of series and shunt resistances, the latter may be internally biased, even though the external voltage across the tandem is zero during EQE or jV measurements. The internal biasing depends on the intensity and spectral content of the illumination. This may lead to the tandem short-circuit current being different from the current of the limiting cell, particularly if the photocurrent collection is voltage-dependent.^27^

Complementary to the optical improvements and bandgap changes, a NiO_*x*_ layer, introduced via wet-chemical deposition of nanoparticles, was added to the HTL layer prior to SAM deposition. Currently, the stability and durability of perovskite-containing devices are being investigated. In the literature, NiO_*x*_ has been used to improve device stability, as it can lead to enhanced charge-carrier dynamics and overall device performance.^28,29^ However, our conjecture is that the aforementioned nonuniformity of the NiO_*x*_ layer allowed halide-ion migration, which has been observed to cause light-induced metastability in perovskite-based PV devices.^30^ Therefore, identifying methods to suppress or stabilize ion migration, as well as further lowering the CIGS bandgap and fine-tuning the bandgap of perovskite, will be part of our focus for future improvements.

## 5 Conclusions

In conclusion, this work presents an independently certified world record for a monolithically grown 2T perovskite/CIGS tandem solar cell with a steady-state performance of 24.6% ± 1.1% on an active area of 1.105 cm^2^, which, according to in-house measurements conducted before shipping to the certification lab, achieved a noncertified PCE of 27%. To achieve this record, variations regarding the top and bottom subcells were implemented. For the coevaporated CIGS bottom subcell, the bandgap depth profile was changed by varying the temperature during the second stage (i.e., from 530 to 490 °C) of the known three-stage process, which brought the minimum bandgap, as calculated from the chemical composition, from about 1.08–1.10 eV to 1.05–1.06 eV. For the spin-coated perovskite top subcell, the solution was changed to aim for a lower bandgap of 1.63 eV (i.e., Cs_0.05_(FA_0.83_MA_0.17_)_0.95_Pb­(I_0.83_Br_0.17_)_3_) to better match the lower bandgap of the CIGS. In addition, the IZO window layer thickness was decreased from 100 to 40 nm, allowing extra photons into the bottom cell and providing better current matching. These modifications resulted in a photogenerated current density increase from 19.26 to 19.88 mA/cm^2^ for the bottom cell and an increase from 18.73 to 20.05 mA/cm^2^ of the top cell. Thus, the overall photogenerated current density, extracted from certified *IV* measurements of the world record tandem, was improved from ∼18.8 to ∼19.3 mA/cm^2^ through bandgap engineering and modification of the IZO window layer.

## Supplementary Material



## Notes

SI.1: Optical simulations; SI.2: thickness measurement of NiO_*x*_ layer; SI.3: chronological *IV* measurements of the perovskite/CIGS tandem; SI.4: perovskite preparation (PDF)
