# Supplementary material for: Improving Perovskite/CIGS Tandem Solar Cells for Higher Power Conversion Efficiency through Light Management and Bandgap Engineering
Source: ACS Appl Mater Interfaces. 2025 Sep 25;17(40):56250–5. doi: 10.1021/acsami.5c15458 (PMC12516680; doi:10.1021/acsami.5c15458)
Supplement: Supplementary file 1 [file am5c15458_si_001.pdf]

Supporting Information

Improving perovskite/CIGS tandem solar cells for higher power conversion efficiency through light management and bandgap engineering

Guillermo Farias-Basulto[1,*], Thede Mehlhop[1], Nicolas J. Otto[2], Tobias Bertram[1], Klaus Jäger[1,3], Stefan Gall[1], Nikolaus Weinberger[4], Rutger Schlatmann[1,2], Iver Lauermann[1], Reiner Klenk[1], Emil List-Kratochvil[1,5,6], Christian A. Kaufmann[1]

1 Helmholtz-Zentrum Berlin für Materialien und Energie GmbH, Hahn-Meitner-Platz 1, 14109 Berlin, Germany

2 HTW Berlin - University of Applied Sciences, Wilhelminenhofstr. 75a, D-12459 Berlin, Germany.

3 Zuse Institute Berlin, Takustraße 7, 14195 Berlin, Germany

4 Universität Innsbruck, Institut für Konstruktion und Materialwissenschaften, Technikerstraße 13, 6020 Innsbruck, Austria

5 Humboldt-Universität zu Berlin, Institut für Physik, Institut für Chemie, Zum Großen Windkanal 2, 12489 Berlin, Germany

6 Center for the Science of Materials Berlin, Zum Großen Windkanal 2, 12489 Berlin, Germany

*Corresponding author

E-mail address: guillermo.farias@helmholtz-berlin.de

SI.1 Optical simulations

Optical simulations were performed with GenPro4 [S1], which is based on the net-radiation method and can handle the propagation of light in layers coherently or incoherently. Further, scattering at rough interfaces can be handled, for example, with ray tracing.

Table S.1: The layer stack used for the optical simulations and the simulated current densities corresponding to the absorption in the layers, which are calculated as e.g. given in Ref. [S2]. Eq. (1). GGI denotes Ga/(Ga + In).

Material		Thickness (nm)	***Simulated* current density *(mA/cm²)* *t=40* nm**	***Simulated* current density *(mA/cm²)* *t=100* nm**
R		-	3.06	3.69
LiF	[S3]	110	0	0
IZO	[S4]	*t* = [40,100]	0.51	1.25
SnO_2	[S5]	20	0.13	0.12
C_{60}	[S5]	23	1.28	1.22
triple cation perovskite	[S5]	550	20.16	19.53
NiO	[S6]	<15	0	0
AZO	[S5]	60	0.19	0.18
i-ZnO	[S7]	40	0.02	0.02
CdS	[S7]	50	0.07	0.07
CIGS (GGI 15%)	[S8]	1,100	19.45	18.86
CIGS (GGI 30%)	[S8]	1,100	1.06	1.03
Mo	[S7]	800	2.75	2.71

Table S.1 shows the detailed layer stack used for the simulations, including the sources of the complex refractive index (n,k) data and the simulated current densities corresponding to the absorption in the layers for the two IZO thicknesses of 40 nm and 100 nm. In the simulations, light propagation in the perovskite, CIGS and Mo layers was treated incoherently, while it was treated coherently in all other layers. The ~ 1 nm LiF interlayer and the self-assembled monolayer on top and bottom of the perovskite layer, respectively, were omitted in the simulations. To account for the bandgap grading in the CIGS layer, we split it into two equally thick layers with Ga/(Ga + In) (GGI) ratios of 15% and 30%, respectively.

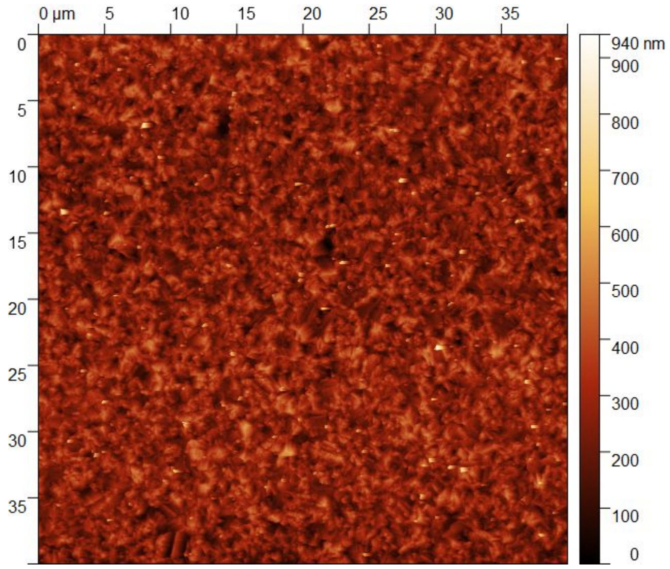

Fig. S.1: Atomic force microscopy picture of a CIGS surface.

The interfaces between the CIGS bottom and the perovskite top absorbers were assumed to have the roughness of a generic CIGS surface, shown in Fig. S.1. In the optical simulation, scattering at these interfaces was treated with ray tracing. All other interfaces were treated as optically flat.

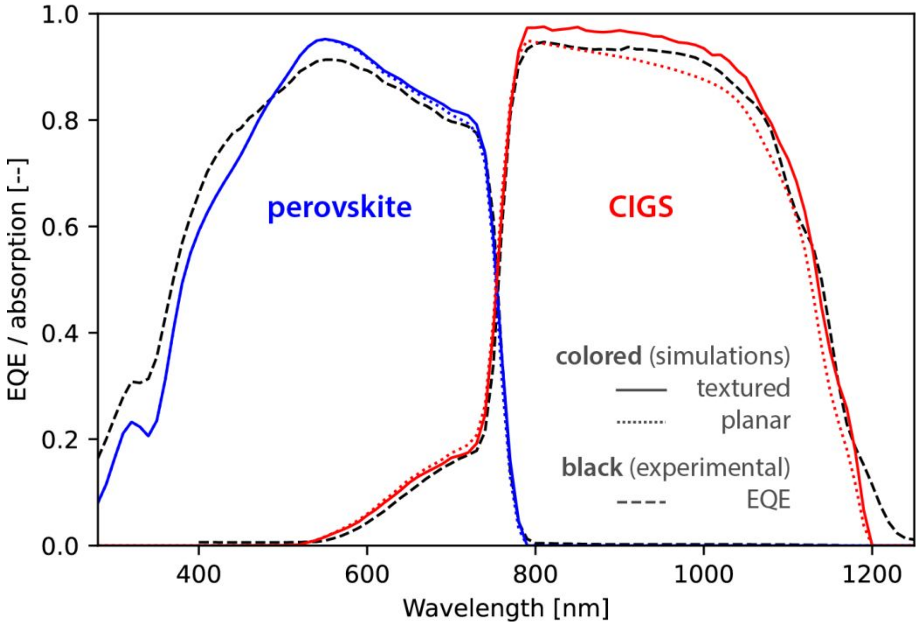

Fig. S.2: Simulated absorption and experimentally measured EQE of perovskite and CIGS sub-cells, respectively. For the simulations, the IZO thickness was set to 40 nm.

Fig. S.2 shows the simulated absorption and experimentally measured EQE of perovskite and CIGS subcells, respectively. For the simulations, the IZO thickness was 40 nm. Simulated and experimental data show excellent agreement close the absorption edge of CIGS around 1100 – 1200 nm. This indicates that using two CIGS layers with different GGI ratios is sufficient to mimic the experimental bandgap grading from an optical point of view. Initially there was an offset between the simulated and measured band edge of perovskite, even though we used an *n,k* data file that was determined for perovskite with the same composition. This might hint to slight deviations in the fabrication processed in the different labs. To get a good match at the perovskite band edge, the *n,k* data was wavelength-shifted by 10 nm, a process that we successfully applied in the past [S8]. To have a good match at the CIGS absorption onset at ~600 nm, we set the perovskite thickness to 550 nm. While this value is thicker than the nominal thickness of 500 nm, the real thickness variations are much larger because of the roughness of the CIGS layer (see Fig. S.1). Figure S.2 shows simulation results for planar and textured CIGS surfaces. For CIGS, the planar case has a good match at 800 nm, which gets worse with increasing wavelength. For the textured case, the simulated absorption curves are slightly above the measured EQE data for large parts of the spectrum. This overestimation can be attributed to imperfect *n,k* data and electrical losses that affect the EQE. Because the textured case resembles the real device architecture better, we consider it for further simulations. As shown in Fig. S.3, changing the NiO thickness from 15 nm to 5 nm does not affect the absorption curves. Overall, the match between experimental and simulated data is sufficient to study the optical effect of varying IZO thickness.

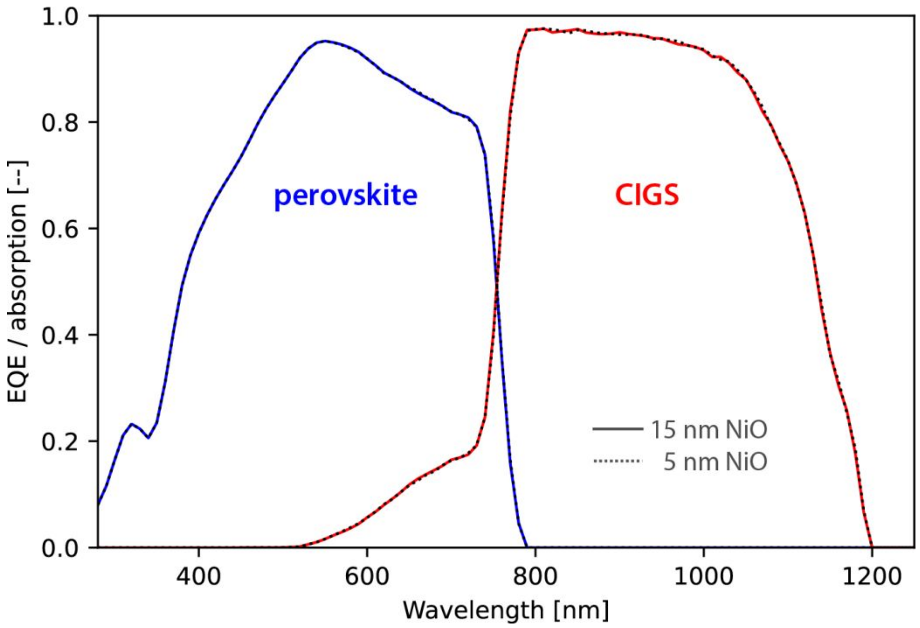

Fig. S.3: Effect of changing the NiO thickness from 15 nm (as in Fig. S.2) to 5 nm. The IZO thickness was 40 nm.

[S1] R. Santbergen *et al.*, IEEE J. Photovolt. **7**, 919–926 (2017).
[S2] K. Jäger *et al.,* Opt. Express **25**, A473–A482 (2017).
[S2] H. H. Li, J. Phys. Chem. Ref. Data **5**, 329 (1976)
[S3] J. Werner *et al.*, Sol. Energy Mater. Sol. Cells **141**, 407 (2015)
[S4] material characterized at HZB
[S5] H. L. Lu *et al.*, Appl. Phys. Lett. **92**, 222907 (2008).
[S6] Delivered with GenPro4
[S7] A. Loubat *et al.,* Applied Surface Science **421**, 643 (2017)
[S8] K. Jäger *et al.*, Opt. Express **25**, A473–A482 (2017).

SI.2 Thickness Measurement of NiOx Layer

Wavelength Dispersive X-ray Fluorescence (WDXRF) measurements were performed with a Rigaku Primus III+ using the fundamental parameter evaluation method. As the evaluation of the NiOx thickness by cross sectional SEM were difficult due to the low thickness the highly sensitive WDXRF was used. For thickness evaluation A stoichiometry of NiO_x with x=1 and a density of 6.72 g/cm³ is assumed. The measurements were performed on full-stack devices after PCE measurement. To evaluate the error the different samples were measured: 1. No NiO_x layer, 2. Record device material containing the solution based (5mg/ml) spin coated NiO_x layer and a device with a 23nm thick sputtered NiO_x layer. For further evaluation of origin of the error of the thickness, the sample without NiOx was measured after HCl treatment (perovskite stack and top layers of CIGS device removed) and after mechanical scratching (CIGS absorber removed). The resulting thickness of all measurements are summarized in Fig. S.4.

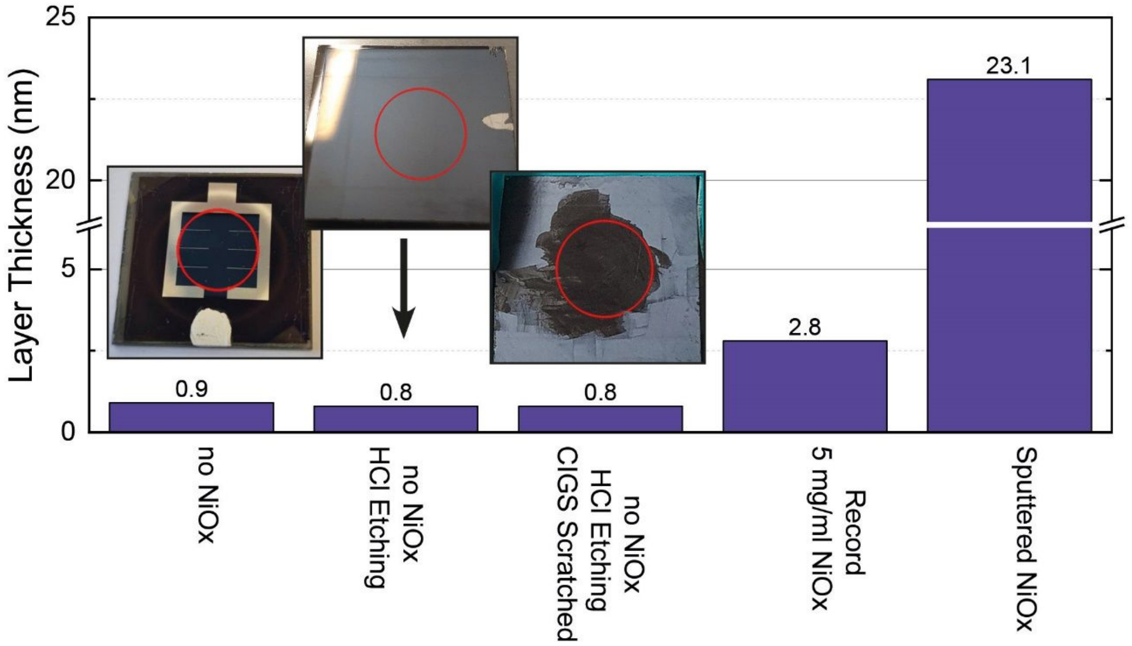

Fig. S.4: Thickness results from WDXRF measurements, including reference samples for accuracy and error evaluation: 1. No NiO_X Layer (1st measurement as it is, 2nd measurement: after HCl etching, 3rd measurement: after HCl etching and mechanical scratching of CIGS absorber); 2. Record cell with spin-coated NiO_X layer; 3. Sputtered 23 nm thick NiO_X layer;

The measurements conclude in a thickness of 2.8nm thickness of the spin-coated NiO_X layer used in the record device. The measurements suggesting an inaccuracy of with an error of 0.8 nm due to impurities in the Molybdenum layer or in the glass substrate and 0.9 nm due to measurement precision.

SI.3 Chronological IV-Measurements of the Perovskite/CIGS Tandem

Initially our in-house measurement presented a PCE of 23.37 area of 1.105 ±0,067 cm² as shown in table S.2. Gradually, the PCE increased with light soaking. The highest PCE of about 27% was obtained after 40 minutes light soak. The most representative IV measurements are shown as example in Fig. S.5.

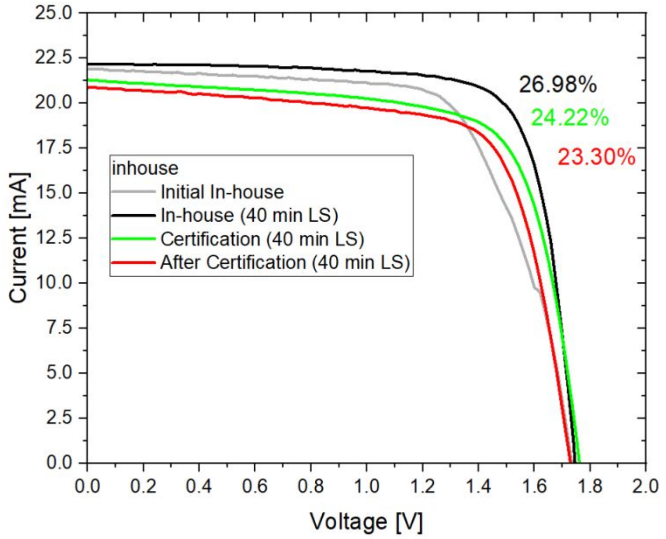

Fig. S.5: IV-measurements of the presented Perovskite/CIGS device.

Table S.2: Table of IV parameters extracted from the IV-curves presented in Fig. S.5, showing the chronological variation in performance of our device.

Measurement	***Light Soak [min]***	***PCE [%]***	***Jsc [mA/cm²]***	***Isc [mA]***	***Voc [V]***	***FF [%]***	***MPP [mW]***
Initial	0	23.37	19.84	21.92	1.74	67.9	25.8
Consecutive LS	3	24.33	19.81	21.89	1.68	73.0	26.9
Consecutive LS	10	25.38	19.94	22.04	1.68	75.6	28.1
Consecutive LS	13	25.57	19.99	22.09	1.69	75.9	28.3
Consecutive LS	16	25.91	20.04	22.15	1.69	76.5	28.6
Consecutive LS	19	26.21	20.06	22.17	1.70	76.8	29.0
Consecutive LS	27	26.79	20.08	22.19	1.73	77.3	29.6
Consecutive LS	31	26.91	20.09	22.20	1.73	77.3	29.7
Consecutive LS	34	26.97	20.08	22.19	1.74	77.3	29.8
Consecutive LS	37	27.01	20.08	22.19	1.74	77.2	29.8
Consecutive LS	40	26.98	20.08	22.19	1.75	77.0	29.8
**Certified (mean)**	**40**	**24.22**	**19.28**	**21.31**	**1.76**	**71.30**	**26.8**
**Steady state record**	**-**	**24.60**	**-**	-	-	-	27.2
After certification	40	23.30	18.88	20.87	1.729	71.35	25.8

SI.5 Perovskite preparation

To prepare the perovskite solution, the chemicals PbI_2, $PbBr_2$, CsI, FAI, and MABr are weighed and placed in separate vials. Then, a 4:1 solution consisting of four parts DMF and one part DMSO is prepared. This solution is added to the vials containing PbI_2 and $PbBr_2$ and mixed on a shaker for at least 3 hours at 900 rpm and 60 °C. CsI is dissolved in pure DMSO. Once PbI_2 and $PbBr_2$ are dissolved, an appropriate amount of the PbI_2 solution is pipetted into the vial containing FAI. Similarly, an appropriate amount of the $PbBr_2$ solution is added to the vial containing MABr. Both mixtures are briefly shaken by hand. The desired concentration of CsI, the FAI-PbI_2 mixture, and the MABr-$PbBr_2$ mixture are then mixed in a third vial to form the final perovskite solution. The solution is filtered through a 0.2 μm PTFE filter before spin-coating. The substrate is blown with nitrogen and placed on

the spin coater, where 100 µl of the perovskite solution is deposited. As the solution does not spread evenly over the substrate, it is spread across the surface with a pipette tip. Care must be taken to only touch the surface of the perovskite liquid film, not the substrate surface. After spreading, the substrate is spun for 5 seconds at 3000 rpm and then for 35 seconds at 3500 rpm. Exactly 25 seconds after the spinning starts, 250 µl of Anisol is evenly deposited onto the substrate from about 0.5 cm. This results in the formation of a perovskite layer about 500-550 nm thick. After spin-coating, the substrate is placed on a 100 °C hot plate for 30 minutes.
